# Supplementary material for: Substrate recruitment via eIF2γ enhances catalytic efficiency of a holophosphatase that terminates the integrated stress response
Source: Proc Natl Acad Sci U S A. 2024 Mar 28;121(14):e2320013121. doi: 10.1073/pnas.2320013121 (PMC10998612; doi:10.1073/pnas.2320013121)
Supplement: Supplementary file 1 — Appendix 01 (PDF) [file pnas.2320013121.sapp1.pdf]

Fig. S1 Dephosphorylation of a non-specific reference substrate [glycogen phosphorylase A (PYGM)] by apo PP1, PPP1R15A core and extended holophosphatases.

(A) Coomassie stained SDS-PAGE PhosTag gels of PYGM^{P} following dephosphorylation in vitro by apo PP1, core or extended holophosphatases. 10 nM PP1, 300 nM G-actin and 400 nM core R15A or extended R15A were incubated for 20 min at room temperature, then mixed with 1uM PYGM^{P} for different time intervals at 30˚C. Shown is a representative of experiments reproduced three times.

(B) The panel displays the mean ± s.d. of the k_{cat}/K_{m} extracted from three independent experiments with the indicated holophosphatases. *P* values for two tailed parametric t-test are shown.

Fig. S2 Flexibility of eIF2 trimer and the N-terminally extended PPP1R15A-containing holophosphatase hint at the difficulty in solving the structure of a dephosphorylation complex of eIF2^{P}.

(*A*) Two and three dimensional reconstructed images of complexes of eIF2^{P} and an extended holophosphatase comprised of human PPP1R15A$^{325\text{-}636}$, PP1A and G-actin. The green volume on the left represents the previously determined cryo-EM structure of PP1^{D64A}/G-actin/DNase I/R15A$^{553\text{-}624}$/eIF2α^{P}-NTD (PDB 7NZM). Two low resolution cryo-EM volumes were obtained from the complex of PP1^{H66K}/G-actin/DNase I/R15A$^{325\text{-}636}$/eIF2α^{P}γ (blue and yellow surface contoured at a similar level) by ab-initio reconstruction in CryoSPARC: the blue is similar to the 7NZM (map correlation is 0.86) and the yellow volume is different and bigger than the former. Below are selected 2D classes generated from the particles classified for these two volumes. Despite the presence on the grid of particles of a size consistent with the eIF2^{P} dephosphorylation complex, we were unable to solve the structure at a meaningful resolution.

(*B*) MD simulations of the eIF2 trimer

Shown is a simulation starting from the highest ranked AFM model of eIF2. The top panel display overlaid three distinct conformations of the eIF2 trimer throughout the simulation trajectory, underscoring the flexibility of the eIF2α-CTD, eIF2β, and eIF2γ, compared to the relatively stable eIF2α-NTD. The middle panels depict the RMSD plots for eIF2α-hinge (residue 184-190) and the composite eIF2α-CTD-eIF2β-eIF2γ. RMSD calculations were performed after aligning the entire trajectory on the eIF2α-NTD, because of its considerable stability (RMSD 2.3 ± 0.3 Å). The cartoon illustrates the potential for the bulky eIF2 lobe comprised of βγ and α-CTD subunits to interfere with the alignment of the flexibly-attached eIF2α^{P}-NTD in the active site, by clashing with the holoenzyme.

(*C*) MD simulations of the extended holophosphatase

MD simulations of the extended holophosphatase highlight the flexibility of the N-terminal extension of PPP1R15A (PPP1R15A$^{331\text{-}554}$). Three specific conformations of the extended holophosphatase are displayed over the course of the simulation trajectory. The components are coloured as indicated and the blue circle represents the PP1 active site. RMSD of PPP1R15A was calculated with respect to PP1 and G-actin proteins. RMSD values reported and computed were specifically for the Cα atoms. Solid lines in plot panels represent the exponential moving average throughout the MD simulations for each respective replicate. The cartoon depicts the largely disordered N-terminal extended PPP1R15A inhibiting substrates engagement.

Fig. S3 AFM prediction and MD simulations of eIF2γ and PPP1R15A complexes.

(*A*) The pLDDT (predicted local distance difference test) and PAE (Predicted Aligned Error) plots for AFM predictions of the human eIF2 trimer in complex with the repeat-containing region of PPP1R15A$^{325\text{-}517}$.

(*B*) The pLDDT and PAE plots for AFM prediction of complexes of human eIF2γ and individual repeats of PPP1R15A: R1$^{331\text{-}376}$, R2$^{377\text{-}420}$, R3$^{421\text{-}466}$ or R4$^{471\text{-}503}$ respectively.

(C) Plots of time-dependent variation of distances between the atoms of human PPP1R15A L^{338} or V^{343} and the indicated residues of human eIFγ throughout 500 ns of an all-atom MD simulations performed using the AFM predicted complex structure of eIF2γ and repeat 1 (PPP1R15A$^{331\text{-}376}$). Shown are three replicates of the simulation.

(*D*) The dynamic interactions between the acidic segment of PPP1R15A's repeat 1 region (PPP1R15A$^{348\text{-}360}$) and the positively charged surface of eIF2γ. The left panel displays the interface interaction captured at time=500 ns from the calculations of replicate 1. The right panel presents a stability analysis based on the count of contacts between PPP1R15A$^{348\text{-}360}$ and the positively charged surface of eIF2γ throughout the MD simulations. The cutoff radius for computing the contact was set at 6 Å.

Fig. S4 Phylogenetic conservation of PPP1R15 repeats and their binding site in eIF2γ.

(*A*) Sequence alignment of repeat 3 of human PPP1R15A (aa. 424-450) with homologues and orthologues. Conserved hydrophobic residues that contact eIF2γ across PPP1R15A, PPP1R15B in mammals and PPP1R15 in other vertebrates and in human herpesvirus 1 are marked by stars.

(*B*) AFM model predicting the ability of human R15A-R3$^{421-467}$ to bind to the same conserved hydrophobic groove in yeast eIF2γ as in human eIF2γ. On the left is an overlaid AFM prediction of human R15A in complex with human eIF2γ (grey) or yeast eIF2γ (colored by pLDDT). The RMSD over 426 pruned atom pairs is 0.48Å. The arrow points at the helix binding in the hydrophobic groove of eIF2γ. On the right are the pLDDT and PAE plots of AFM prediction of the human R15A-R3$^{421-467}$ and yeast eIF2γ complex.

Fig. S5 The eIF2γ-contacting Phe and Trp residues of PPP1R15A contribute to eIF2^{P} dephosphorylation in vitro, by reduced PPP1R15A$^{420-636}$-containing holophosphatase confined to repeats 3 and 4

(*A*) Coomassie stained SDS-PAGE PhosTag gels of dephosphorylation reactions of the core substrate (eIF2α^{P}_NTD, upper panel) or the physiological substrate (eIF2^{P} trimer, lower panel) by a holophosphatase comprised of the wildtype PPP1R15A$^{420-636}$ (reduced to repeats 3 and 4) or its 2X FW>AA mutant counterpart (F428A, W432A, F479A, W482A), G-actin and PP1A. Shown is a representative of experiments reproduced three times.

(*B*) Graphic display of the mean ± s.d. of the k_{cat}/K_m extracted from all experimental points with the indicated substrates and holophosphatases. *P* values for two tailed parametric t-test are shown.

Fig. S6 PPP1R15A contacts with eIF2γ contribute to ISR termination in cells transfected with mammalian expression plasmids encoding the counterpart to the PPP1R15A$^{325-636}$ used in vitro.

Dual channel flow cytometry plots of untreated and thapsigargin (Tg)-treated CHOP:GFP transgenic CHO-K1 cells transfected with mCherry alone or human PPP1R15A (wild type or mutants as indicated) with mCherry fused to their C-termini.

Fig. S1

A

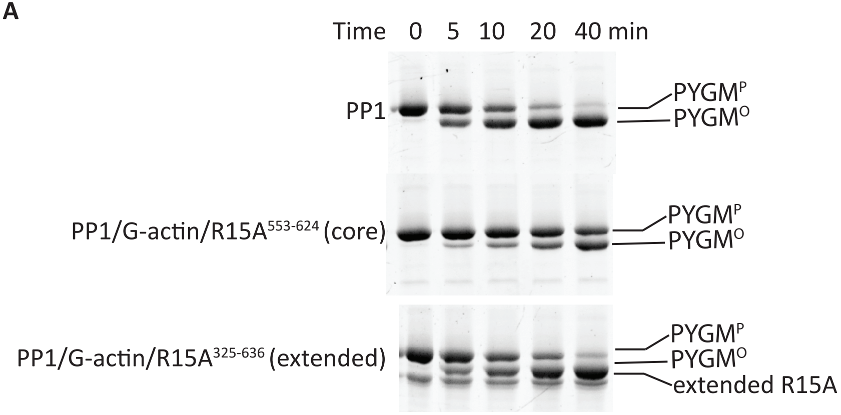
Time 0 5 10 20 40 min
PP1
PP1/G-actin/R15A553-624 (core)
PP1/G-actin/R15A325-636 (extended)
PYGMP
PYGMO
extended R15A

B

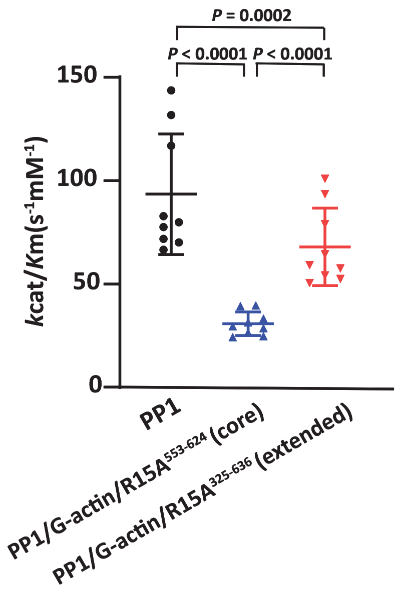
P = 0.0002
P < 0.0001
P < 0.0001
150
100
50
0
kcat/Km(s-1mM-1)
PP1
PP1/G-actin/R15A553-624 (core)
PP1/G-actin/R15A325-636 (extended)

Fig. S2

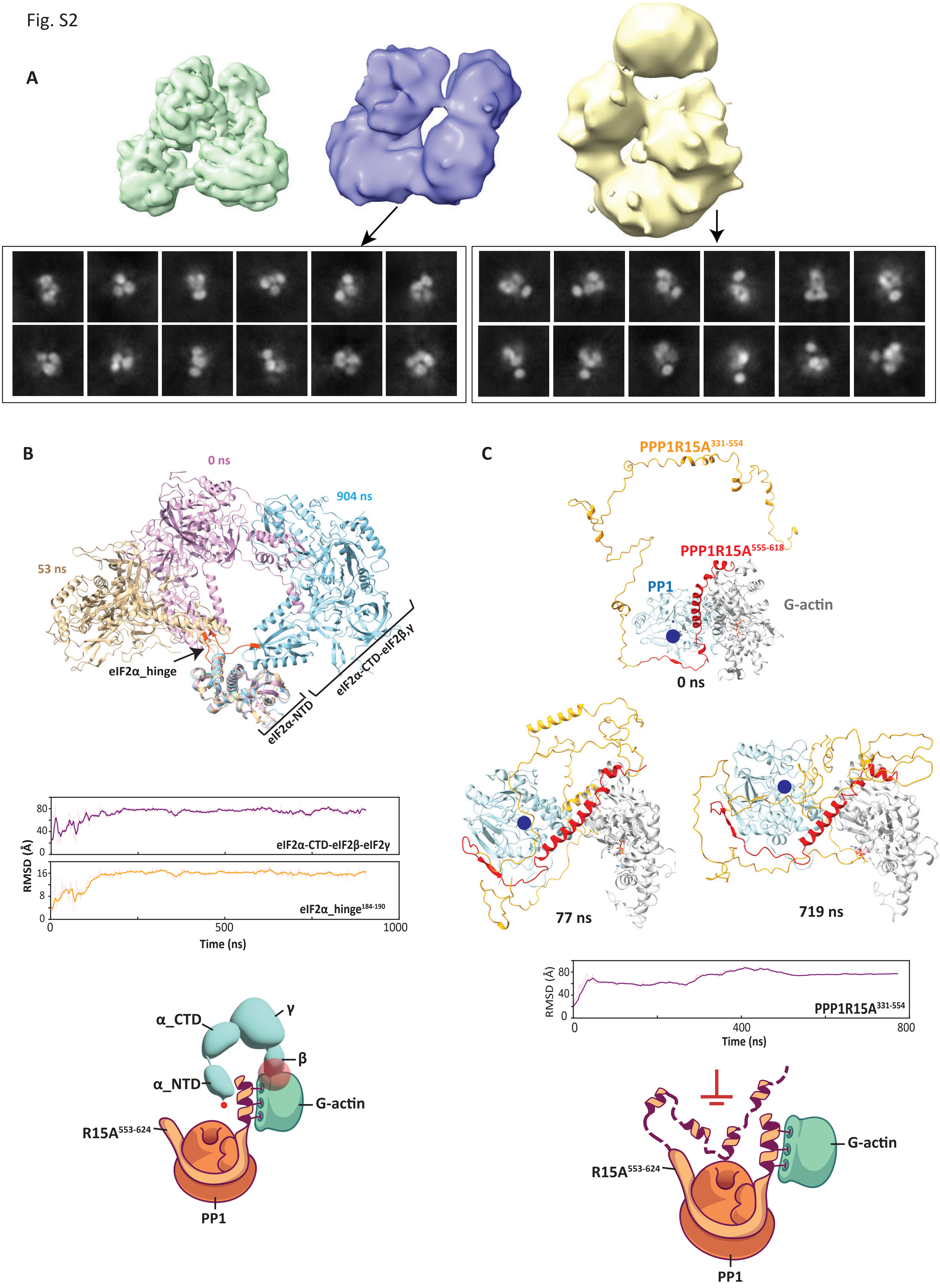
A
B
0 ns
904 ns
53 ns
eIF2α_hinge
eIF2α-NTD
eIF2α-CTD-eIF2β,γ
RMSD (Å)
eIF2α-CTD-eIF2β-eIF2γ
eIF2α_hinge184-190
Time (ns)
α_CTD
γ
β
α_NTD
G-actin
R15A553-624
PP1
C
PPP1R15A331-554
PPP1R15A555-618
PP1
G-actin
0 ns
77 ns
719 ns
RMSD (Å)
PPP1R15A331-554
Time (ns)
G-actin
R15A553-624
PP1

Fig. S3

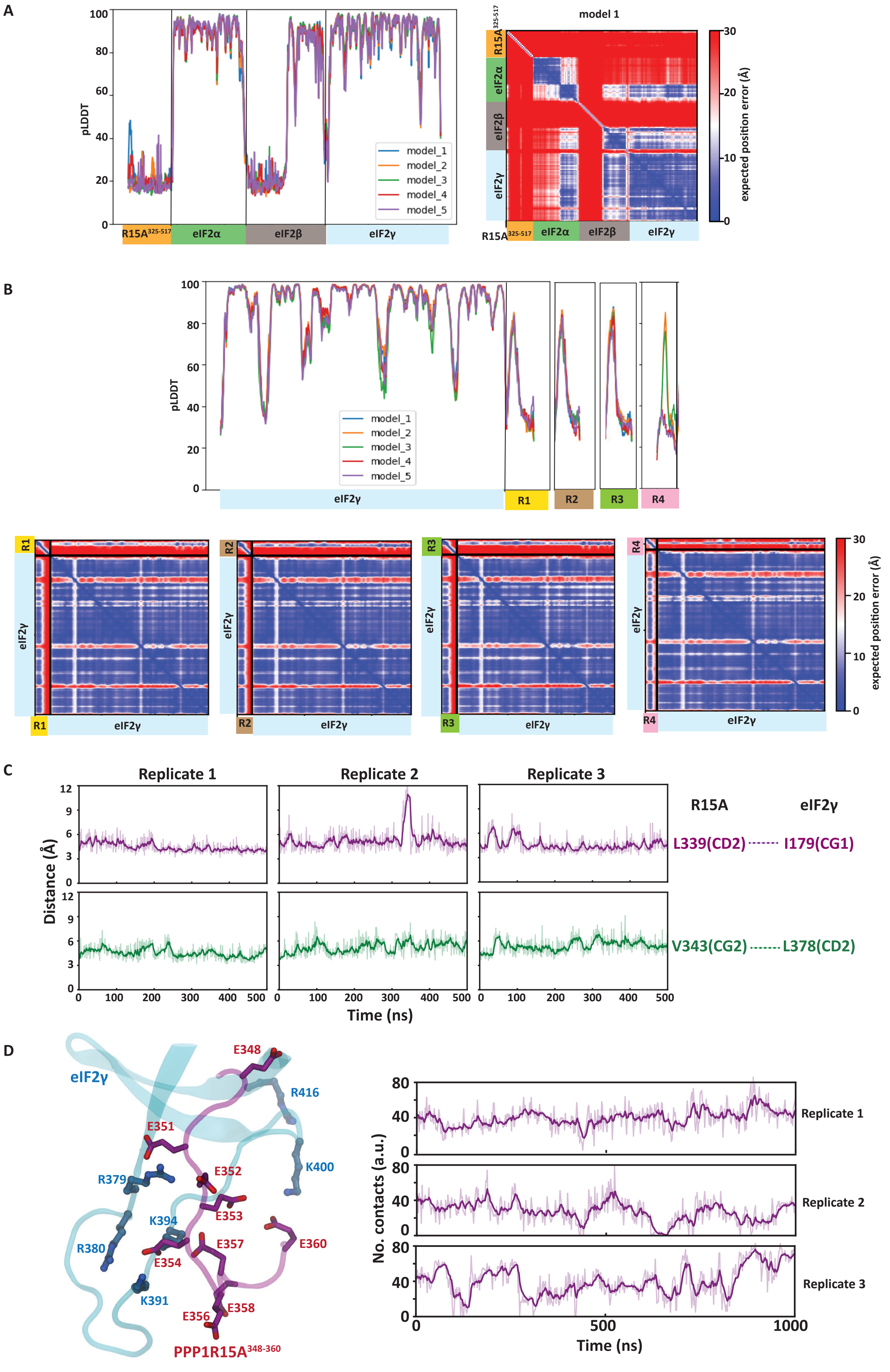
A
pLDDT
model_1
model_2
model_3
model_4
model_5
R15A325-517
eIF2α
eIF2β
eIF2γ
model 1
expected position error (Å)
B
pLDDT
R1
R2
R3
R4
expected position error (Å)
C
Replicate 1
Replicate 2
Replicate 3
Distance (Å)
Time (ns)
R15A
eIF2γ
L339(CD2) I179(CG1)
V343(CG2) L378(CD2)
D
eIF2γ
E348
R416
E351
K400
R379
E352
E353
K394
R380
E354
E357
E360
K391
E356
E358
PPP1R15A348-360
No. contacts (a.u.)
Time (ns)
Replicate 1
Replicate 2
Replicate 3

Fig. S4

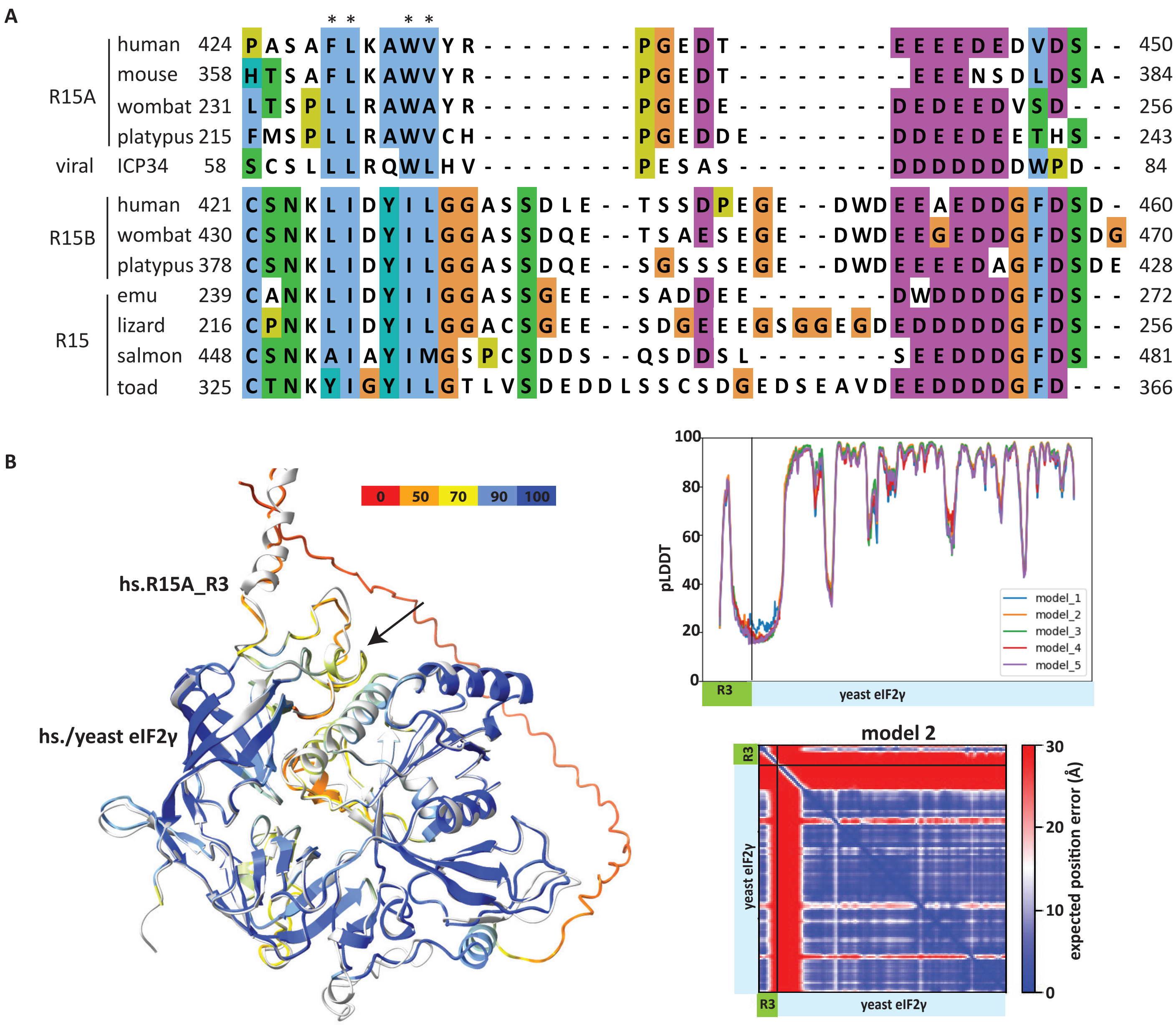

A
R15A
viral
R15B
R15
human 424 PASAFLKAWVYR---------PGEDT----------EEEEDEDVDS-- 450
mouse 358 HTSAFLKAWVYR---------PGEDT-----------EEENSDLDSA- 384
wombat 231 LTSPLLRAWAYR---------PGEDE----------DEDEEDVSD--- 256
platypus 215 FMSPLLRAWVCH---------PGEDDE---------DDEEDEETHS-- 243
ICP34 58 SCSLLLRQWLHV---------PESAS----------DDDDDDDWPD-- 84
human 421 CSNKLIDYILGGASSDLE--TSSDPEGE--DWDEEAEDDGFDSD- 460
wombat 430 CSNKLIDYILGGASSDQE--TSAESEGE--DWDEEGEDDGFDSDG 470
platypus 378 CSNKLIDYILGGASSDQE--SGSSSEGE--DWDEEEEDAGFDSDE 428
emu 239 CANKLIDYIIGGASSGEE--SADDEE-------DWDDDDGFDS-- 272
lizard 216 CPNKLIDYILGGACSGEE--SDGEEEGSGGEGDEDDDDDGFDS-- 256
salmon 448 CSNKAIAYIMGSPCSDDS--QSDDSL-------SEEDDDGFDS-- 481
toad 325 CTNKYIGYILGTLVSDEDDLSSCSDGEDSEAVDEEDDDDGFD--- 366
B
0
50
70
90
100
hs.R15A_R3
hs./yeast eIF2γ
pLDDT
model_1
model_2
model_3
model_4
model_5
R3
yeast eIF2γ
model 2
expected position error (Å)

Fig. S5

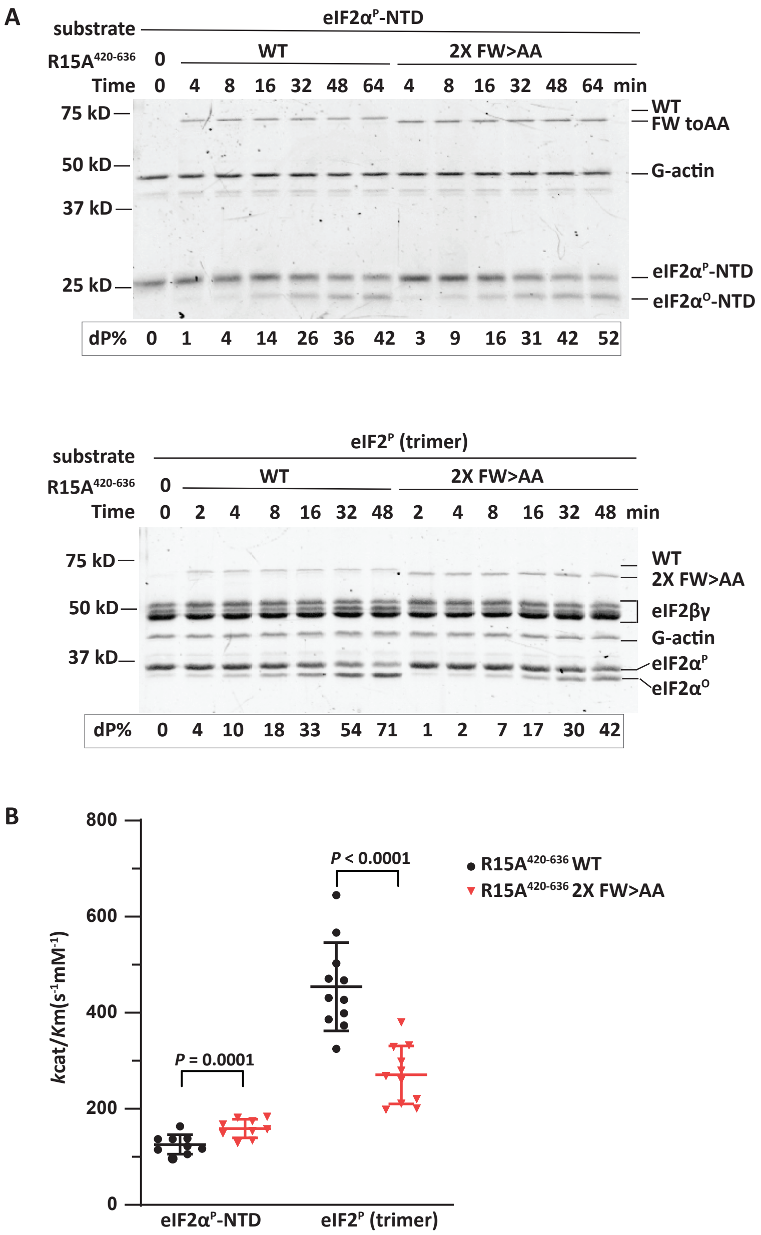
A
substrate
eIF2α^P-NTD
R15A^420-636
0
WT
2X FW>AA
Time 0 4 8 16 32 48 64 4 8 16 32 48 64 min
75 kD
50 kD
37 kD
25 kD
WT
FW toAA
G-actin
eIF2α^P-NTD
eIF2α^O-NTD
dP% 0 1 4 14 26 36 42 3 9 16 31 42 52
substrate
eIF2^P (trimer)
R15A^420-636
0
WT
2X FW>AA
Time 0 2 4 8 16 32 48 2 4 8 16 32 48 min
75 kD
50 kD
37 kD
WT
2X FW>AA
eIF2βγ
G-actin
eIF2α^P
eIF2α^O
dP% 0 4 10 18 33 54 71 1 2 7 17 30 42
B
800
600
400
200
0
kcat/Km(s-1mM-1)
P = 0.0001
P < 0.0001
R15A^420-636 WT
R15A^420-636 2X FW>AA
eIF2α^P-NTD
eIF2^P (trimer)

Fig. S6

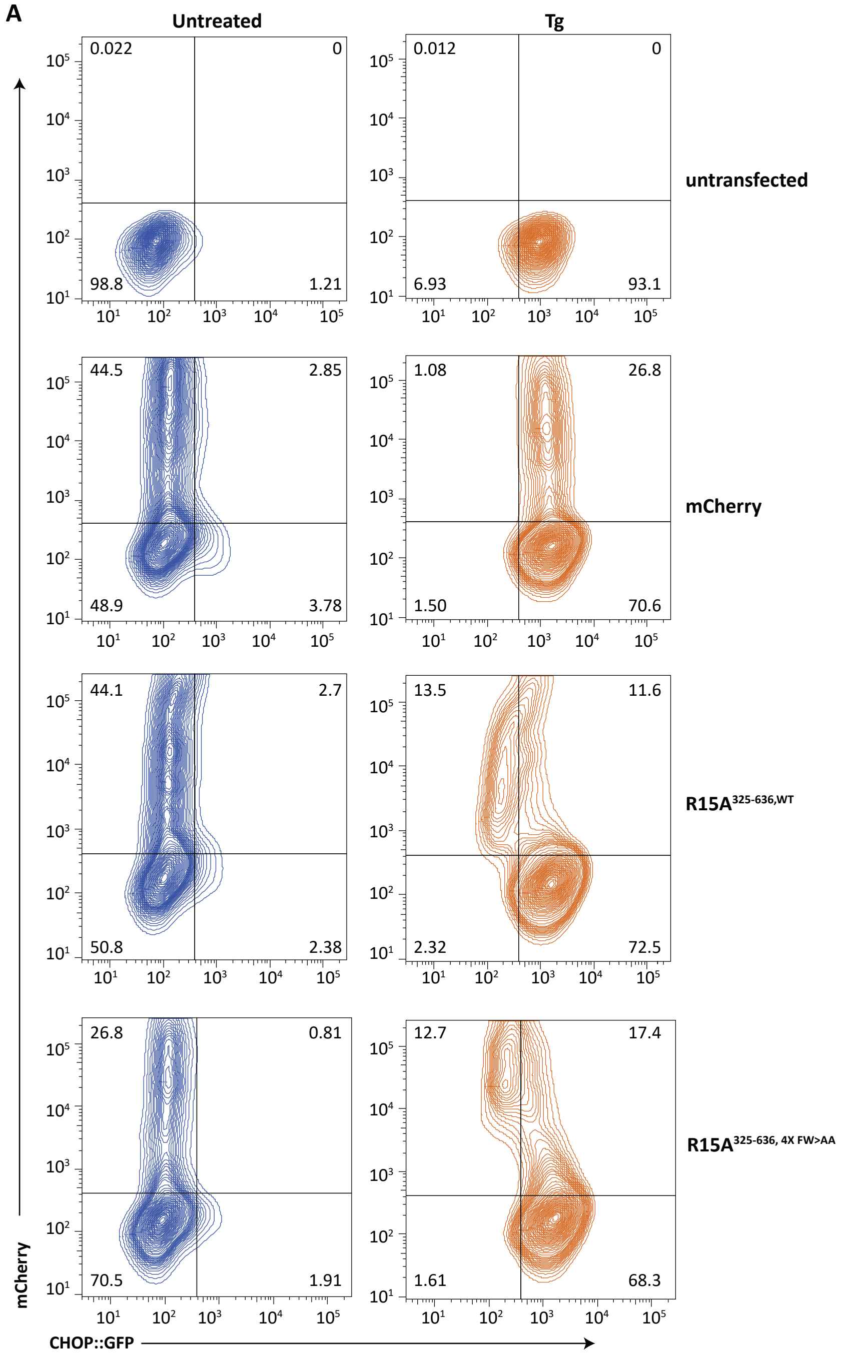
A
Untreated
Tg
untransfected
mCherry
R15A325-636,WT
R15A325-636, 4X FW>AA
0.022
0
98.8
1.21
0.012
0
6.93
93.1
44.5
2.85
48.9
3.78
1.08
26.8
1.50
70.6
44.1
2.7
50.8
2.38
13.5
11.6
2.32
72.5
26.8
0.81
70.5
1.91
12.7
17.4
1.61
68.3
mCherry
CHOP::GFP

Table S1: List and description of plasmids used in this study.

ID	Plasmid name	Description	Reference	Correspon ding figures
UK1920	huPPP1R15A_533_624_malE_pGEX _TEV_AviTag	Bacterial-expression of N-term Avi-Tagged human GADD34 533-624 C-term MBP	PMID 28447936	1B, 3B, S1A
UK2668	SENP2_364-589_pET-28a MP9	Bacterial expression of human SUMO3 protease SENP2 active fragment		1B, S1, 2D, S2A, 3, S4,
UK2731	heIF2a_2-187_pSUMO3	encodes H6-SUMO3-SER_hueIF2a_2-187	PMID 34625748	1B, 3B, S1A
UK759	pRK793 His6_TEV(S219V)_Arg	His-TEV(S219V)-Arg from pRK793. Gift to HH from Wang Lab		3A, 2D
UK2937	hu PPP1R15A_325-636-MBP_pSUMO3	Bacterial-expression of human GADD34 325-636 C-term MBP	this paper	1B, 3B, S1A
UK2940	rPP1A_7-300_pSUMO3	H6Smt3-tagged Rabbit PP1A_7-300	this paper	1B, 3B, S1A
UK2960	rPP1A_7-300_H66K_pSUMO3	H66K based on uk2940 construct	this paper	S2A
UK2996	hR15A_420-636-MBP_pSUMO3	Bacterial expression of PEST hR15A core extended by PEST repeat 3 & 4	this paper	S4
UK2997	hR15A_420-466_C_TEV-MBP-pSUMO3	Bacterial expression of human PPP1R15A repeat 3 with a C-term Cys for labelling	this paper	2B, 2D 3A
UK3083	hR15A_420-636-MBP_F428A, W432A, F479A, W482A_pSUMO3	GADD34_PEST R3 and R4 with F428A, W432A, F479A, W482A mutations	this paper	S4
UK3098	hu PPP1R15A_420-466_F428A_W432A_C_TEV_MBP	Mutant version of UK2997	this paper	3A
UK3133	hu PPP1R15A_325-636_4X FW>AA-MBP_pSUMO3	Bacterial-expression of 4X FW>AA mutanthuman GADD34 325-636 C-term MBP	this paper	3B
UK3136	H6SUMO3-IF2A_Cdc123_MBP-TEV-IF2G	Polycistronic bacterial expression of eIF2a, eIF2g and CDC123	this paper	2D, S2A
UK3188	hR15A_420-452_C-pSUMO3	Trimmed version of UK2997 (for co-crystalisation with eIF2)	this paper	2D
UK1657	huPPP1R15A 1-673-pEGFP N1 (WT)	mammalian expression of wildtype human GADD34 fused at C-term to EGFP	PMID 25774599	4C and 4D
UK2661	huGADD34-pEGFP N1 (R556E)	mammalian expression of PP1 interacting mutant (R556E) human GADD34 fused at C-term to EGFP	this paper	4C and 4D
UK3218	huPPP1R15A 1-673-pEGFP N1 (4X FW>AA)	mammalian expression of human GADD34 in which conserved Phe and Trp in the four repeats were converted to Ala fused at C-term to EGFP	this paper	4C and 4D
UK3238	huPPP1R15A 1-673-pEGFP N1 (4X FW>AA; R556E)	mammalian expression of human R556E (PP1-interacting mutant GADD34 in which conserved Phe and Trp in the four repeats were converted to Ala fused at C-term to EGFP (F338A; W342A; F385A; W389A; F428A; W432A; F479A; W482A)	this paper	4D
UK3165	huPPP1R15A_1-673_WT_mCherry_3XFLAG	mammalian expression of wildtype human GADD34 fused at C-term to mCherry tagged with 3XFLAG	this paper	4A
UK3166	huPPP1R15A_1-673_4X FW>AA_mCherry_3XFLAG	mammalian expression of human GADD34 in which conserved Phe and Trp in the four repeats were converted to Ala fused at C-term to mCherry tagged with 3XFLAG	this paper	4A
UK3202	pCEFL_mCherry_3XFLAG	Parent plasmid of UK3165, UK3166, UK3178	this paper	4A
UK3178	huPPP1R15A_546-621_WT_mCherry_3XFLAG	mammalian expression of wildtype human GADD34 core fused at C-term to mCherry tagged with 3XFLAG	this paper	4A
UK3099	FLAG_huPPP1R15A_325-636_4X	mammalian expression of N-terminally FLAG-tagged extended human GADD34 in which conserved Phe and	this paper	S5

	FW>AA 4X_mCherry V2	Trp in the four repeats were converted to Ala fused at C-term to mCherry		
UK3107	FLAG_huPPP1R15A_325-636_WT_mCherry V3	mammalian expression of N-terminally FLAG-tagged extended wild-type human GADD34 in which conserved Phe and Trp in the four repeats were converted to Ala fused at C-term to mCherry	this paper	S5

Table S2: X-ray data collection and refinement statistics.

	**eIF2αγ/R15A[420-452] complex**
**Data collection**	
Synchrotron stations	DLS i24
Space group	$P4_12_12$
a,b,c (Å)	120.59, 120.59, 158.26
α, β, γ (°)	90.00, 90.00, 90.00
Resolution (Å)	41.17-3.35 (3.35-3.62)[a]
Rmerge	0.271 (2.618)[a]
<I/σ(I)>	7.2 (1.1)[a]
CC1/2	0.996 (0.521)[a]
No. of unique reflections	17421 (3497)[a]
Completeness, %	99.9 (100.0)[a]
Redundancy	12.3 (12.3)[a]
**Refinement**	
Rwork/Rfree	0.241/0.299
No. of atoms (non H)	4218
Average B-factors	141
RMS Bond lengths (Å)	0.01
RMS Bond angles (°)	1.911
Ramachandran favoured region (%)	96.13
Ramachandran outliers (%)	0
PDB code	8QZZ

[a] Values in parentheses are for the highest resolution shell.

Table S3 Key resource table

Reagent type (species) or resource	Designation	Source or reference	Identifiers	Additional information
Cell line (*Cricetulus griseus*)	CHO-K1, CHOP::GFP, XBP1:turquoise	PMID: 26673894	S21	
Cell line (*Human*)	HEK 293 T	ATCC	CRL 3216	
Cell line (Human)	U20S lacking GADD34	Unpublished	U2OS_GADD34Δ	Generated using CRISPR-Cas9 to target exon I of PPP1R15A, creating an 8 base pair deletion that resulted in a premature stop codon at nucleotide position 579 and loss of protein expression
Commercial assay or kit	MycoAlert (TM) Mycoplasma Detection Kit	Lonza	LT07-118	
Purified protein	Phosphorylase A (purified from rabbit muscle in the phosphorylated form on serine 15)	Sigma (Cat No. P1261)	PYGM	
Chemical compound, drug	Thapsigargin	MERCK-milipore	586005	
Chemical compound, drug	Sodium arsenite	MERCK	1.06277	
Antibody	Rabit polyclonal serum to eIF2a N-terminal domain	PMID: 25774600	anti-eIF2a (Lab name NY1308)	WB (1:3000)
Antibody	Mouse monoclonal to protein phosphatase 1 catalytic subunit (all isotypes)	Gift of Dr. Mathieu Bollen, University of Leuven, PMID: 20671031	anti-PP1	WB (hybridoma supernatent, undiluted)
Antibody	Goat polyclonal anti-rabbit IgG (H+L); IRDye 800CW Secondary Antibody	Li-Cor	Cat. #: 926–32211	WB (1:2000)
Antibody	Goat polyclonal anti-mouse IgG (H+L); IRDye 680CW Secondary Antibody	Li-Cor	Cat. #: 926–68070	WB (1:2000)

Antibody	Mouse Anti-human G3BP1	Becton Dickinson BioScience	Clone 23/G3BP (RUO	ICC (0.5 µg/mL)
Antibody	Goat Anti-Mouse IgG H&L (Alexa Fluor® 594)	Abcam	ab150116	ICC (0.25 µg/mL)
Software, algorithim	Prism 10	GraphPad		Data analysis
Software, algorithim	Harmony High-Content Imaging and Analysis Software	Revvity		Used in SG assay
Software, algorithim	FlowJo	Becton Dickinson BioScience		FACS analysis
Software, algorithim	CCP4i2 (1.1.0)	PMID: 21460441		Used in X-ray crystallography
Software, algorithim	Phenix (1.20.1-4487)	PMID: 20124702		Used in X-ray crystallography
Software, algorithim	WARP (v.1.0.9)	PMID: 31591575		Used in Cryo-EM data processing
Software, algorithim	CryoSPARC (v.4.3.0)	PMID: 28165473		Used in Cryo-EM data processing
Software, algorithim	AlphaFold2 (versions 2.2.2 onwards)	PMID: 34265844		Used for Structure prediction
Software, algorithim	AlphaFold Multimer	doi: https://doi.org/10.1101/2021.10.04.463034		Used for Structure prediction
Software, algorithim	UCSF ChimeraX (v.1.6.1)	PMID: 37774136		Used for structural graphics
Software, algorithim	PyMOL (v.1.3)	Schrödinger, LLC		Used for structural graphics
Software, algorithim	EPU	Thermo Fisher's data acquisition software		Used in Cryo-EM data collection
Software, algorithim	GDA	DLS-developed generic data acquisition software		Used in X-ray crystallography
